# OP32 Cost-Effectiveness Analysis Of Prenatal Surgery For Myelomeningocele In The Private Healthcare System In A Middle-Income Country

**DOI:** 10.1017/S0266462325100901

**Published:** 2025-12-29

**Authors:** Luciana Przysiezny, Rafael Bruns, Layssa Oliveira, Mariana Fachi

## Abstract

**Introduction:**

Spina bifida (myelomeningocele [MMC]) is the most common congenital anomaly with permanent disability. Traditional treatment consists of repairing the defect itself and correcting hydrocephalus (via shunt surgery), when present, within the first 48 hours after birth. Since 2011, fetal surgery to correct MMC has become an impactful treatment alternative, with better short-term results, both neurological and cognitive. However, cost remains a limiting factor for guidelines and health policy changes.

**Methods:**

Fetal surgery (the alternative) was compared with neonatal surgery (the comparator) in a cost-utility analysis. The perspective of the study was from supplementary health, with direct cost data extracted from the public database of the National Supplementary Health Agency (Agência Nacional de Saúde Suplementar). The time horizon considered was 10 years, with a discount rate of five percent. The effectiveness measure was quality-adjusted life years (QALYs). Eligibility, utility, and outcome data were obtained from medical literature. The model used was Markov, with coupled decision tree. Subsequently, probabilistic and deterministic sensitivity analyses were carried out, and the cost-effectiveness acceptability curve (CEAC) was calculated.

**Results:**

The results indicated that the incremental cost-effectiveness ratio (ICER) of fetal surgery for MMC was dominant, with savings of BRL162,956 (USD26,972.80) per QALY gained. Probabilistic sensitivity analysis showed uncertainty, with 87.3 percent of points agreeing with base case results, but with some dispersion. CEAC showed that fetal surgery is more likely to be cost effective, regardless of the willingness-to-pay threshold. Through deterministic univariate sensitivity analysis, the variation in the cost of the shunt and complications due to prematurity by ±20 percent presented the greatest variations in the ICER, showing the possibility of an increase in cost.

**Conclusions:**

This study demonstrated that prenatal repair generated cost savings, being cost effective, with an ICER of minus BRL162,956 (USD26,972.80) per QALY gained. This is the first study conducted at national level comparing fetal and neonatal myelomeningocele surgery. Although this new approach has been associated with higher costs, these additional costs translate into better long-term results, which could justify changes in health policies.

